# Technology-Aided Spatial Cues, Instructions, and Preferred Stimulation for Supporting People With Intellectual and Visual Disabilities in Their Occupational Engagement and Mobility: Usability Study

**DOI:** 10.2196/33481

**Published:** 2021-11-17

**Authors:** Giulio E Lancioni, Nirbhay N Singh, Mark F O'Reilly, Jeff Sigafoos, Gloria Alberti, Valeria Chiariello, Francesca Campodonico, Lorenzo Desideri

**Affiliations:** 1 University of Bari Bari Italy; 2 Augusta University Augusta, GA United States; 3 University of Texas at Austin Austin, TX United States; 4 Victoria University of Wellington Wellington New Zealand; 5 Lega F. D'Oro Research Center Osimo Italy; 6 University of Bologna Bologna Italy

**Keywords:** technology, smartphone, motion sensors, intellectual disability, visual impairments, occupational engagement, mobility, mobile phone

## Abstract

**Background:**

Persons with severe or profound intellectual disability and visual impairment tend to be passive and sedentary, and technology-aided intervention may be required to improve their condition without excessive demands on staff time.

**Objective:**

This study aims to extend the assessment of technology-aided interventions for supporting functional occupational engagement and mobility in 7 people with intellectual disability and visual impairment and to use a technology system that is simpler and less expensive than those previously used.

**Methods:**

The technology system involved a Samsung Galaxy A10, 4 Philips Hue indoor motion sensors, and 4 mini speakers. Within each session, the participants were to collect 18 objects (ie, one at a time) from 3 different areas (stations) located within a large room, bring each of the objects to a central desk, and put away each of those objects there. For each object, the participants received verbal (spatial) cues for guiding them to the area where the object was to be collected, a verbal instruction (ie, request) to take an object, verbal (spatial) cues for guiding them to the central desk, a verbal instruction to put away the object collected, and praise and preferred stimulation.

**Results:**

During baseline, the frequency of responses completed correctly (objects collected and put away independently) was 0 or near 0. During the intervention phase (ie, with the support of the technology setup), the frequency increased for all participants, reaching a mean of almost 18 (out of 18 response opportunities) for 6 participants and about 13 for the remaining participant. The mean session duration ranged from 12 to 30 minutes.

**Conclusions:**

A program, such as the one used in this study, can be useful in promoting occupational engagement and mobility in persons with intellectual disability and visual impairment.

## Introduction

### Background

People with severe to profound intellectual disability tend to be passive and sedentary when not provided with direct supervision from staff or caregivers [1-6]. Passivity and sedentariness may become even more serious when people present with a combination of intellectual disability and visual impairment [7-12]. To modify this negative situation, efforts are required to design intervention strategies suitable for promoting occupational engagement and mobility (ie, indoor walking), that is, for (1) providing people with a chance of meaningful actions and (2) increasing their opportunities for physical exercise and environmental stimulation [13-19].

To be effective, intervention strategies are expected to support people in critical areas such as (1) the appropriate use of objects (eg, taking and putting away objects at the right places), (2) spatial orientation for moving from one place to another (eg, to reach and use the objects), and (3) engagement motivation (eg, willingness to walk, orient to the cues, and use objects appropriately) [13,15,20-22]. To ensure sufficient support in the aforementioned areas and promote functional occupation and mobility independent of staff, intervention strategies need to rely on the support of technology [11,16,22-25].

One of the intervention strategies that rely on technology [11] was designed to provide the participants with (1) spatial (verbal) cues to help them reach different destinations where objects had to be collected, (2) preferred stimulation for reaching the destinations, (3) spatial (verbal) cues to transport the objects collected to a container, and (4) preferred stimulation for reaching the container and putting away the objects transported. The technology at the basis of this intervention strategy was expressly built for the study and included (1) electronic boxes with optic sensors (one box and sensor at each of the destinations) used for presenting spatial cues, detecting the participant’s arrival, and delivering preferred stimulation and (2) a remote electronic control unit used to regulate the functioning of the boxes and sensors.

Another intervention strategy relying on technology [16] differs from the one described earlier in two main aspects. First, it also provides the participants with instructions for the use of objects at the destinations (ie, *take an object* or *put away the object*). Second, the technology components on which this strategy was based are all commercially available (as opposed to being specifically built) and include a number of smartphones, mini speakers, and portable light sources that are combined in clusters. A cluster (ie, a smartphone, mini speaker, and light source) was available at each destination to be reached for taking or putting away objects.

The aforementioned technology-aided intervention strategies were reported to be effective in helping participants reach independent occupation and mobility. Notwithstanding the encouraging results, additional research is warranted to (1) verify whether these results can be replicated across studies and (2) upgrade (improve on) the technology previously used. Successful replications would allow one to make more definite statements about the overall impact and generality of technology-aided intervention strategies [26,27]. Upgrading the technology may be critically important in view of the fact that (1) the technology system used by Lancioni et al [11] was specifically built for the purpose of the study and thus is not easily accessible and rather expensive and (2) the technology system used by Lancioni et al [16] involved several clusters of smartphones, mini speakers, and light sources; thus, it may be considered fairly complex and expensive.

### Objectives

This study was conceived as a systematic replication effort whose main purpose was to (1) extend the assessment of technology-aided strategies to support independent functional occupation and mobility in people with intellectual disability and visual impairment, and (2) evaluate a relatively simple, commercially based technology system, which would be cheaper and more accessible than the systems used for the aforementioned strategies. The new technology system was based on the use of a single smartphone combined with motion sensors and mini speakers and was assessed with 7 participants.

## Methods

### Participants

Table 1 identifies the 7 participants (representing a convenience sample) by their pseudonyms and reports their chronological age, their visual and motor impairments, and the age equivalents for their daily living skills on the second edition of the Vineland Adaptive Behavior Scales [28,29]. The participants’ chronological age ranged from 14 to 54 years. One of the participants (Alec) had functional residual vision, which allowed him to see immediate objects and obstacles. The remaining 6 participants were completely blind. One of these 6 (Davis) also presented with severe motor impairment and required the use of a wheelchair. The Vineland age equivalents for daily living skills (personal domain) ranged from 1 year and 7 months (Davis) to 3 years and 2 months (Maggie and Alec). All participants attended rehabilitation and care centers. Their psychological records indicated that their level of intellectual disability had been estimated to be within the severe or severe to profound range, but no specific tests were applied for their assessment.

**Table 1:** Participants’ pseudonyms, chronological ages, visual and motor impairments, and Vineland age equivalents for Daily Living Skills, Personal Sub-domain (DLSP).

Participants’ pseudonyms	Chronological age (years)	Visual and motor impairments	Vineland age equivalents^a,b^ (DLSP)
Maggie	23	Blindness	3; 2
Lauryn	14	Blindness	3; 1
Alec	40	Severe visual impairment with functional residual vision	3; 2
Bill	35	Blindness	2; 8
Jay	22	Blindness	2; 9
Davis	44	Blindness and severe motor impairment requiring the use of a wheelchair	1; 7
Erin	54	Blindness	2; 11

^a^The age equivalents are based on the Italian standardization of the Vineland scales.

^b^The Vineland age equivalents are reported in years (number before the semicolon) and months (number after the semicolon).

The participants were included in the study based on the following criteria: First, they could follow auditory spatial cues and reach the destinations indicated by these cues. Second, they could respond to simple verbal instructions concerning taking and putting away objects. Third, they were known to enjoy (eg, to show behaviors such as alerting and smiling in relation to) forms of preferred environmental stimulation such as music, praise, and voices of favorite family or staff members. Thus, it was thought that the use of such stimuli contingent on their response performance could be a motivating (reinforcing) event for strengthening and maintaining such performance. Fourth, activities involving mobility and object use were considered important examples of functional occupation to counter the participants’ sedentariness and passivity. Moreover, the participants were reported to be comfortable (eg, to show no signs of fatigue or anxiety) when engaging in such types of activities under staff supervision. Fifth, staff supported the study (whose purpose and required technology had been presented to them in advance), as they thought that an effective technology-aided intervention could have clearly positive implications for the participants’ activity engagement within the daily context.

Although the availability of preferred stimuli during the study sessions gave reason to believe that the participants might enjoy their involvement in the study, there was no reliable way to determine their assent to be involved. Thus, their legal representatives were asked to sign a consent form on their behalf before the start of the study. The study complied with the 1964 Helsinki Declaration and its later amendments and was approved by an institutional ethics committee.

### Setting, Activities, Sessions, and Research Assistants

Quiet rooms of the centers that the participants attended constituted the setting for the study sessions. An activity consisted of collecting 18 objects (ie, one at a time) from 3 different areas (stations) located within a large room, transporting each of the objects to a central desk, and putting away each of those objects there (ie, depositing each object into a specific container available on the desk). Activities could vary across sessions in terms of the objects to be collected, transported, and put away. The objects could involve kitchen tools, food or drink items, and other simple materials for daily use. For each activity response (ie, object to collect, transport, and put away), the technology system provided (1) verbal cues for guiding the participant to an area with objects to be collected, (2) a verbal instruction (request) to take an object, (3) verbal cues for guiding the participant to the central desk, (4) verbal instruction to put away the object collected, and (5) praise and preferred stimulation. Sessions consisted of the time required for the participants to complete an activity (ie, collect and put away 18 objects). Sessions could also be interrupted before the activity was completed (ie, if a 30-minute time limit had elapsed or the research assistant’s guidance had occurred for 4 consecutive activity responses). Research assistants, who were responsible for implementing the sessions and recording the participants’ responses, had experience in using technology-aided interventions for people with intellectual disabilities and other disabilities.

### Technology System

The technology system used during the intervention phase of the study involved (1) a Samsung Galaxy A10 with an Android 10.0 operating system that was equipped with Amazon Alexa, MacroDroid, and Philips Hue apps; (2) 4 Philips Hue indoor motion sensors; (3) a Philips Hue Bridge and Philips Hue smart bulb working via Bluetooth; (4) a 4G Long-Term Evolution Wi-Fi router; and (5) 4 Bluetooth mini speakers. The Philips Hue Bridge, Philips Hue smart bulb, and Philips Hue app were used to ensure the functioning of the Philips Hue sensors.

The sensors were box-like devices of 5.5 cm width and 3.5 cm height. One of the sensors was placed in front of a central desk to which the participants were to transport the objects collected from 3 different stations in the workroom (setting). The other 3 sensors were placed in front of the 3 stations (one sensor per station). The Bluetooth mini speakers were placed on the desk and at the stations. Any activation of a sensor by the arrival of a participant was detected through the Amazon Alexa app. This app transmitted the arrival message to the smartphone via MacroDroid.

**Figure 1:**
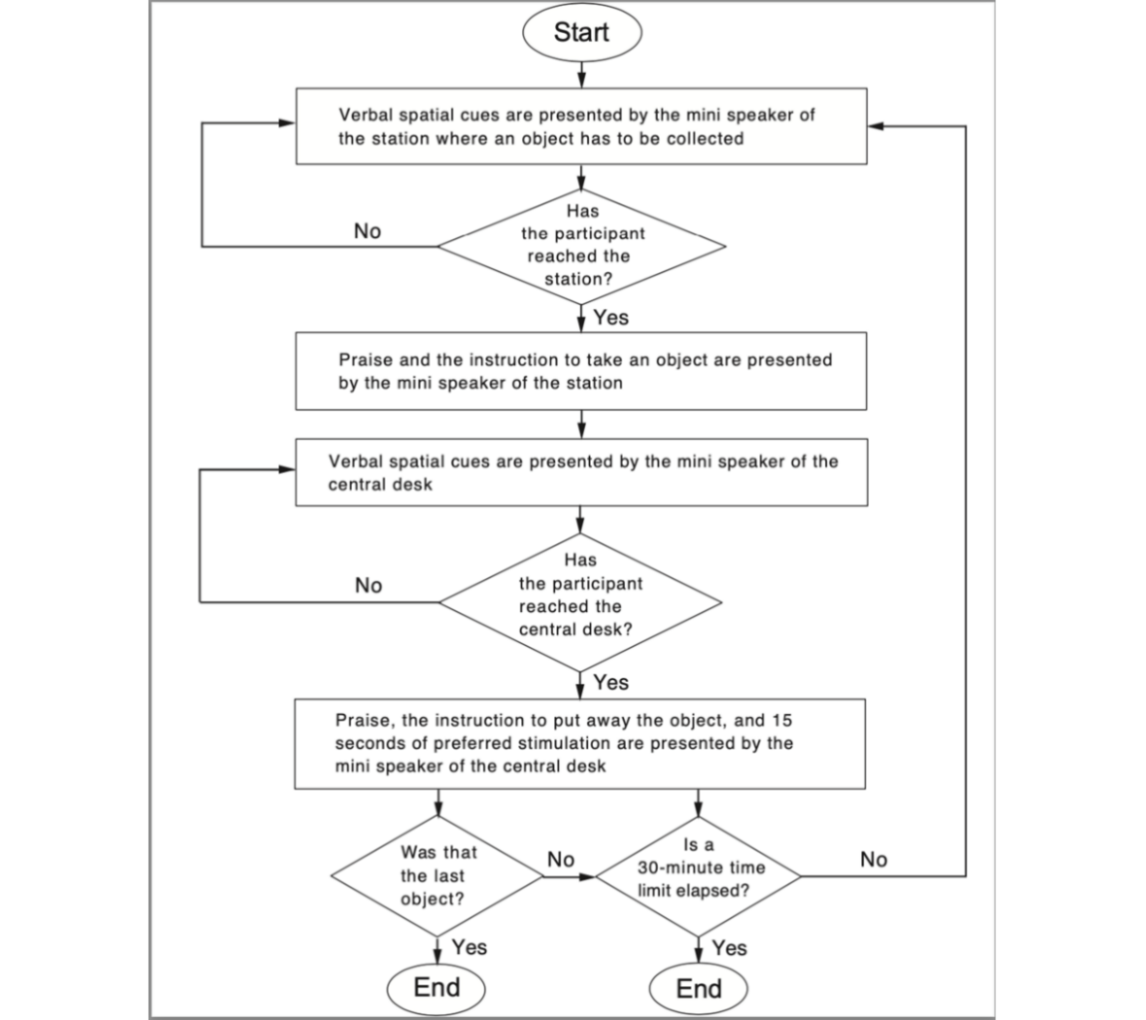
The flowchart summarizes the working of the technology system.

Figure 1 summarizes the working of the technology system. Switching on the system (ie, starting a session) resulted in the mini speaker of the first station to be reached being activated and starting to call the participant (ie, 1-word or 2-word calls that could involve the participant’s name) at intervals of about 5 seconds. The calls served as spatial orientation cues and encouragement to help the participant reach the station. As soon as the participant reached the station (ie, was detected by the sensor before that station and in turn by the Alexa and MacroDroid apps), the mini speaker available at the station presented verbal praise and the instruction to take an object. After a 4-second interval from the instruction, the mini speaker of the central desk started calling the participant, as described above. In response to this sequence of inputs, the participant was expected to take an object and transport it to the central desk. Once the participant reached the central desk and was detected by the sensor there, the mini speaker available at the desk presented praise, the instruction to put away the object, and 15 seconds of preferred stimulation (eg, a 15-second segment of a preferred song or music). At the end of the stimulation, the mini speaker of the next station to be reached was activated (ie, started to call the participant). When the participant arrived, the speaker presented praise and the instruction to take an object, as described above. The same conditions were in use for the other objects that the participant was to collect and transport to the central desk. The session continued until (1) the system had provided the support (ie, spatial cues, instructions, praise, and preferred stimulation) for completing 18 responses, that is, for collecting, transporting, and putting away 18 objects or (2) a 30-minute period had elapsed, whichever came first.

To prevent accidental errors, the system had only one sensor and one speaker functioning at a time during the session; that is, the sensor and speaker of the destination (station or central desk) the participant was to reach. If the participant reached the correct destination, the sensor available there was triggered and the system enacted the programmed events as described earlier. If the participant reached a different destination, the system simply ignored that presence (ie, did not deliver praise, instruction, or preferred stimulation). Meanwhile, the mini speaker of the correct destination continued to call the participant.

### Experimental Conditions

#### Design and General Procedures

The study was conducted according to a nonconcurrent multiple baseline design across participants [30]. In line with the design requirements, the participants were scheduled to receive different numbers of baseline sessions (ie, between 5 and 9) without the support of the technology system. These sessions were followed by intervention sessions, which were carried out with the support of the technology system. In total, 92 to 124 intervention sessions were available for the different participants, with the numbers changing across participants in relation to their availability. Video recordings of the sessions were viewed by a study co-ordinator who was in charge of supervising (providing feedback to) the research assistants to ensure procedural fidelity [31].

#### Baseline

Before the start of a baseline session, the research assistant guided the participant physically and verbally to the different stations where groups of about 10 objects were available. Then, the research assistant (1) accompanied the participant to the proximity of the central desk (as it would occur during the intervention sessions with the technology system) and (2) presented the participant with the instruction to *Go and take an object.* If the participant remained inactive or failed to make progress for 30 to 40 seconds, the research assistant intervened with guidance (ie, guided the participant to take an object from one of the stations, transport the object to the central desk, and put it away in a container available there). This was followed by a new instruction to *Go and take an object*. The research assistant’s guidance was used as described earlier. The session continued until the participant had responded to all the 18 instructions scheduled or until 4 consecutive responses had occurred with the research assistant’s guidance. Session interruption after 4 consecutive guidance instances was used to minimize frustration following repeated failures.

#### Intervention

The intervention phase was introduced by 2 or 3 practice sessions to familiarize the participants with the technology system’s support. During these sessions, the research assistant’s guidance could be used to facilitate the participants’ successful use of such support, even though all participants were known to have the prerequisites for managing such support (ie, for using the cues and responding to the instructions) independently. The regular sessions that followed the practice sessions did not involve the research assistant’s guidance. The research assistant would only accompany the participant to the proximity of the central desk and switch on the technology system to get the sessions started.

Once switched on, the system worked as described earlier (in Technology System; Figure 1). Specifically, the system presented spatial cues, instructions, praise, and preferred stimulation with regard to each of the 18 objects the participant was scheduled to collect and put away within a session that did not exceed a 30-minute limit. The objects were collected from 3 different stations. As in the baseline, each station typically contained 10 objects.

### Measures

The measures were (1) responses completed correctly and (2) session duration. During baseline, a response was completed correctly if the participant reached a destination, took an object (or 2 objects), transported the object to the central desk, and put the object into the container independently, following the initial research assistant’s instruction to *Go and take an object*. During the intervention, a response was completed correctly if the participant displayed the performance sequence mentioned earlier with the support of the technology system (in Technology System; Figure 1). The first measure (ie, responses completed correctly) was recorded by the research assistants who implemented the sessions. The second measure was recorded by (1) the smartphone during the intervention (ie, the smartphone logged the time elapsed from the delivery of the first instruction to the delivery of the last stimulation event at the central desk) and (2) the research assistants during baseline. Interrater agreement was checked in more than 20% of the sessions of each participant on the first measure and all baseline sessions on the second measure by having a reliability observer join the research assistant to record the data. The percentage of agreement on the first measure (computed for each session by dividing the number of responses for which research assistant and reliability observer reported the same *correct* or *incorrect* score by the total number of responses and multiplying by 100%) ranged from 92 to 100, with means exceeding 98 for all participants. The percentage of interrater agreement on the second measure (computed by dividing the number of sessions for which the reported durations differed by <1 minute by the total number of sessions and multiplying by 100%) was 100.

### Data Analysis

The participant’s data for the two measures (ie, responses completed correctly and session duration) are reported in graphic form. To simplify the graphic display, the data were summarized into blocks of sessions (ie, each data point reported in the graphs represents a mean session frequency or a mean session duration computed over a block of sessions). The Kolmogorov-Smirnov test [32] was to be used to analyze the differences between the baseline and intervention frequencies of responses completed correctly for any participant whose data in the two phases presented some level of overlap. In reality, no overlaps were observed.

### Ethical Approval and Informed Consent

Approval for the study was obtained from the Ethics Committee of the Lega F. D’Oro, Osimo, Italy. All procedures performed were in accordance with the ethical standards of the institutional and/or national research committee and with the 1964 Helsinki Declaration and its later amendments or comparable ethical standards. Written informed consent for the participants’ involvement in the study was obtained from their legal representatives.

## Results

**Figure 2:**
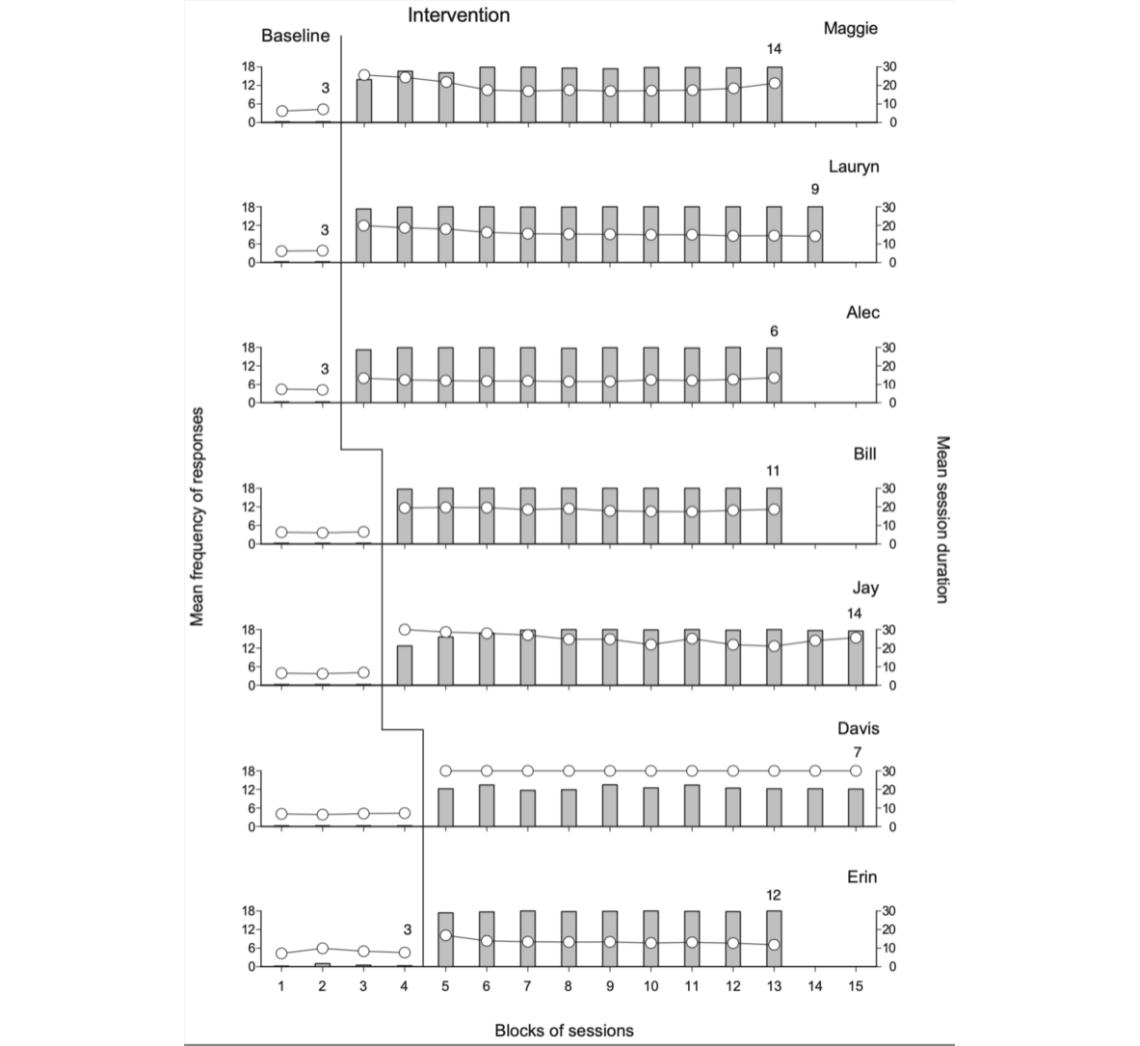
The 7 panels report the participants’ mean frequency of responses completed correctly and mean session duration over blocks of baseline and intervention sessions. The bars represent the mean frequency of responses completed correctly per session over blocks of 2 sessions during the baseline and 10 sessions during the intervention. The circles represent the mean session duration for the same blocks of sessions. Baseline and intervention blocks with different numbers of sessions (ie, appearing at the end of the baseline or the intervention phase) are marked with a numeral that indicates how many sessions they include.

The 7 panels of Figure 2 report the participants’ mean frequency of responses completed correctly and mean session duration over blocks of baseline and intervention sessions. The bars represent the mean frequency of responses completed correctly per session over blocks of 2 sessions during the baseline and 10 sessions during the intervention. The circles represent the mean session duration for the same blocks of sessions. Baseline and intervention blocks with different numbers of sessions (ie, appearing at the end of the baseline or the intervention phase) are marked with a numeral that indicates how many sessions they include. The practice sessions occurring at the start of the intervention phase are not reported in the figure.

During baseline, the frequency of responses completed correctly was 0 for all participants except for Erin, whose level was close to 0. Indeed, all baseline sessions were interrupted after 4 consecutive responses had required guidance from the research assistant. The participants’ mean session duration ranged from 6 to slightly over 8 minutes. During the intervention phase (ie, with the support of the technology system), the mean frequency of responses completed correctly increased for all participants. Six of them (Maggie, Lauryn, Alec, Bill, Jay, and Erin) showed mean frequencies ranging between approximately 13 (Jay) and close to 18 (Bill) during the first block of sessions and approaching 18 during the following blocks of sessions. The mean session duration for these 6 participants varied between approximately 12 (Alec) and 23 minutes (Jay). Session interruptions (ie, after a 30-minute period had elapsed) occurred for Maggie and Jay almost exclusively during the first block of sessions. The intervention data of the remaining participant (Davis) showed a mean frequency of nearly 13 responses completed correctly per session and a mean session duration of 30 minutes. The differences between Davis’ and the other participants’ data are because Davis carried out the responses by propelling his wheelchair. This condition increased the time he needed for each response, and consequently, he could manage only slightly more than two-thirds of the responses scheduled for the sessions before the sessions were interrupted (ie, before the 30-minute time limit was reached).

The absence of overlaps between the baseline and the intervention data in terms of responses correctly completed was seen as clear evidence of the difference between the 2 phases and consequently of the effectiveness of the technology system in promoting correct responses. In light of this evidence, the use of the Kolmogorov-Smirnov test was considered superfluous.

## Discussion

### Principal Findings

The data suggest that the technology system used during the study was effective in supporting the performance of all 7 participants. These results (1) confirm previous data on the feasibility of helping people with intellectual and visual disabilities to independently manage occupational engagement involving mobility and object use through the support of technology-aided programs and (2) add to those data, as a new and relatively simple (commercially based and less expensive) technology system was used to successfully support the participants’ performance [11,14,16,22]. In light of the above, several considerations may be made.

First, given the participants’ baseline performance, which reflected their persistent difficulties in managing occupational engagement, orientation, and mobility, the results of the intervention phase may be considered relevant [11,21,22,33,34]. During the intervention, in fact, the participants managed constructive occupation as well as orientation and mobility with no need for staff supervision. Mobility (via ambulation or self-propelled wheelchair) can be seen as an important component of the results as it represents a form of physical exercise that may have beneficial health-related effects for people like the participants of this study who tend to be largely sedentary [11,35-38].

Second, the technology-based support ensuring the aforementioned results included 3 main components (ie, the calls from the stations or central desk that were to be reached, the instruction to take or put away an object, and the praise or praise and preferred stimulation), which were known to be suitable for the participants (in Participants). The calls were instrumental in helping the participants find the right destinations and may have served as a form of prompt fostering engagement and reducing breaks in performance [11,16]. The instructions may have been critical to ensure that the participants always knew what they were to do, thus avoiding uncertainty and errors [11,14,34]. Praise and preferred stimulation may have been instrumental in motivating the participants’ performance through the sessions and possibly their satisfaction with the sessions [39,40]. Anecdotal reports suggest that the participants showed behaviors such as smiles and vocalizations in connection with the preferred stimulation events.

Third, the technology system used in this study represents a relatively simple and practical tool compared with the systems used previously (ie, systems that relied on specifically built technology devices or on clusters of smartphones, mini speakers, and light sources) [11,16,22]). The cost of the present technology system may be estimated at about US $600 (ie, approximately US $150 for the Samsung smartphone, US $200 for the 4 Philips Hue sensors, US $100 for the 4 mini speakers, and US $150 for the Philips Hue Bridge, the Philips Hue smart bulb, and the 4G Long-Term Evolution Wi-Fi router). Although this cost is significant, one may argue that the present technology system (1) can be one of the few options available to enable people with intellectual and visual disabilities to manage independent occupation and mobility and (2) is fairly easy to operate for personnel in charge of the sessions and friendly for the participants [41-44]. The main obstacle rehabilitation professionals may encounter in accessing such a technology system is represented by the fact that it is not a ready-made (off-the-shelf) tool but needs to be set up through the aforementioned commercial components.

Fourth, research assistants were employed to conduct the study sessions. However, in view of the fact that the technology system seems rather easy to operate, one might envisage regular staff being directly responsible for managing the daily use of the technology and carrying out the sessions. Direct staff responsibility would foster their commitment to maintain the results obtained and an increased likelihood of intervention continuity over time [45,46].

### Limitations

Several limitations of the study should be noted. The first limitation concerns (1) the relatively small number of participants involved in the study and (2) the fact that the participants represented a convenience sample. This limitation makes it difficult to draw conclusive statements about the overall potential and usability of the technology system being evaluated. Direct and systematic replication studies will be essential to determine the strength and reliability of such a system and investigate parallel versions and upgrades of it to improve its suitability and impact [26,27].

The second limitation concerns the lack of assessment of the participants’ satisfaction with the technology system and sessions. Although their successful performance over time suggests that the praise and preferred stimulation available for responding were adequate to motivate their performance, checking their mood during the sessions may add relevant information. Checks might be conducted by recording any behavior that could be representative of happiness and satisfaction (eg, smiles and vocalizations) during the sessions [47-49].

The third limitation concerns the absence of social validation of the technology and its impact. Although staff expressed support for the technology system and its programmed use before the start of the study (Participants), it would be important to determine their opinion as to what the study managed to achieve. Such an assessment (social validation) could be carried out by (1) showing staff a few segments of the sessions carried out with the participants and (2) seeking their ratings of those segments and the technology used in terms of perceived efficacy, friendliness, and applicability [22,50,51].

The fourth limitation is the lack of generalization and maintenance assessments. On the basis of the characteristics of the system, one might reasonably expect successful generalization across settings and intervention agents to occur, as the conditions responsible for the participants’ performance (ie, spatial orientation cues, instructions, and praise and preferred stimulation) would remain identical regardless of the setting and the intervention agents involved [39]. With regard to maintenance, the perspectives might be closely tied to whether preferred (motivating) stimulation continues to be available. In essence, participants are likely to maintain their positive performance if the stimulation they receive contingent on it is enjoyable for them [39,40].

### Conclusions

In conclusion, one might argue that the technology system assessed in this study can be effective in helping people with intellectual and visual disabilities manage independent occupational engagement involving mobility and object use. Although the data appear quite encouraging, general statements about the system and its usability cannot be made until new research has successfully addressed the aforementioned limitations of this study. Future research may also explore the possibility of simplifying the present system through the use of new (cheaper) commercial technology components so that the new version could be more easily arranged and more readily applicable in daily contexts.
